# Bayesian multisource data integration for explainable brain-behavior analysis

**DOI:** 10.3389/fnins.2022.1044680

**Published:** 2022-10-28

**Authors:** Rong Chen

**Affiliations:** Department of Diagnostic Radiology and Nuclear Medicine, University of Maryland School of Medicine, Baltimore, MD, United States

**Keywords:** Bayesian network, brain-behavior analysis, explainable AI, Bayesian inference, data fusion

## Abstract

Different data sources can provide complementary information. Moving from a simple approach based on using one data source at a time to a systems approach that integrates multiple data sources provides an opportunity to understand complex brain disorders or cognitive processes. We propose a data fusion method, called Bayesian Multisource Data Integration, to model the interactions among data sources and behavioral variables. The proposed method generates representations from data sources and uses Bayesian network modeling to associate representations with behavioral variables. The generated Bayesian network is transparent and easy to understand. Bayesian inference is used to understand how the perturbation of representation is related to behavioral changes. The proposed method was assessed on the simulated data and data from the Adolescent Brain Cognitive Development study. For the Adolescent Brain Cognitive Development study, we found diffusion tensor imaging and resting-state functional magnetic resonance imaging were synergistic in understanding the fluid intelligence composite and the total score composite in healthy youth (9–11 years of age).

## 1. Introduction

A central topic in neuroscience is understanding the association between the brain and behavior in normal and diseased states. Neuroimaging provides a non-invasive tool to study brain structure and function *in vivo* and is a powerful tool for brain-behavior analysis. A brain characterization framework is referred to as a data source (“source” here means the source or cause of a particular data feature). A data source can be an imaging method such as resting-state functional magnetic resonance imaging (fMRI); or it can be a kind of feature from an imaging method, for example, structural MRI can generate four data sources: volume, thickness, surface, and curvature. Most existing neuroimaging studies focus on a single data source. Many brain disorders are complex diseases. It's highly unlikely that one source will be able to fully capture the brain disorder. Different sources can provide complementary information. Moving from a simple approach based on using one source at a time to a systems approach that integrates multiple sources provides an opportunity to identify composite neuroimaging biomarkers for brain disorders.

Explainable AI (XAI) aims to develop AI algorithms in which the processes of action (e.g., predictions or recommendations) can be easily understood by users. Explainable models enable users to understand and appropriately trust the developed models. Interpreting the decision-making process of models in the biomedical domain is especially important.

We propose a method, called Bayesian Multisource Data Integration (BAMDI), to model the interactions among data sources and behavioral variables. BAMDI generates a representation from a data source and associates the representation with behavioral variables. The generated representation is referred to as embedding. The embedding is a set of vectors. Each vector is referred to as a factor. BAMDI has the following features. First, it centers on brain-behavior analysis. Many data integration methods focus on generating shared representation and cannot answer the question of how cross-source interactions are related to the behavior (Geenjaar et al., 2021; Zhang et al., 2022). In contrast, BAMDI represents interactions among different sources and behavioral variables as a Bayesian network. Brain-behavior analysis is the core of BAMDI. Second, BAMDI is an XAI method. Unlike some black-box methods, the Bayesian network generated by BAMDI is transparent and easy to understand. We use Bayesian inference to understand how the perturbation of a factor is related to the behavioral change.

Various Bayesian fusion methods for neuroimaging data have been proposed. Wei et al. developed a Bayesian fusion method to provide informative (empirical) neuronal priors—derived from dynamic causal modeling of electroencephalogram data—for subsequent dynamic causal modeling of fMRI data (Wei et al., 2020). Kang et al. proposed a Bayesian hierarchical spatiotemporal model to combine diffusion tensor imaging (DTI) and fMRI data (Kang et al., 2017). This method uses DTI-based structural connectivity to construct an informative prior for functional connectivity estimation. A parametric Bayesian multi-task learning based approach is developed to fuse univariate trajectories of neuroimaging features across subjects (Aksman et al., 2019). This Bayesian method fuses neuroimaging data across subjects, instead of modalities. Different from the above methods, the proposed method centers on modeling the interactions among data sources and behavioral variables with Bayesian network modeling, an XAI method.

In what follows, we first describe the overall design of BAMDI and its constituent modules. Following this, we applied BAMDI to simulated data to establish face validity. In other words, to ensure that the proposed scheme can recover the known brain-behavior mappings used to generate synthetic data. After this, we applied BAMDI to empirical data—from a publicly available databank—to characterize the relationship between MRI data from children, and their behavioral phenotypes as assessed with a battery of standard neurocognitive instruments.

## 2. Methods

### 2.1. Background

One of the foundations of BAMDI is Bayesian network modeling (Pearl, 1988; Koller and Friedman, 2009). A Bayesian network B={G,Θ} is a probabilistic graphical model, where G={V,E} is a directed acyclic graph. A node *X* in V is a random variable in the problem domain. E is the edge set. A parent node of *X* is a node from which there exists a directed edge to *X*. The parent set of *X* is denoted by *pa*(*X*). The local distribution is the conditional distribution *P*(*X*|*pa*(*X*)). The full specification of local distribution is the parameterization of the network. **Θ** is the set of parameters. The joint distribution can be represented compactly: $P\left(V\right)=\prod_{i}P\left(X_{i}|pa\left(X_{i}\right)\right)$. In BAMDI, we adopt the discrete Bayesian network representation and all nodes are discrete variables because the discrete Bayesian network can represent any kind of distribution among discrete variables and has high representation power. In a discrete Bayesian network, *P*(*X*_*i*_|*pa*(*X*_*i*_)) is a conditional probability table. For node *X*_*i*_, the conditional probability θ_*ijk*_ = *P*[*X*_*i*_ = *k*|*pa*(*X*_*i*_) = **j**] is the probability that node *X*_*i*_ assumes state *k* when the parent set of *X*_*i*_ assumes state **j**. If *X*_*i*_ has no parents, then θ_*ijk*_ is the marginal distribution of *X*_*i*_. **Θ** = {θ_*ijk*_} is the parameters of discrete Bayesian network.

Bayesian network structure learning aims to learn G. Bayesian network parameter learning is the process to estimate **Θ**. Score-based structure learning methods use a score that reflects how well the data support the structure and search for a structure that can optimize the fitness score. For discrete Bayesian networks, a widely used score is the Bayesian Dirichlet equivalent uniform (BDeu) score (Heckerman et al., 1995).

Bayesian network inference performs queries about probability distribution once some evidence about variables is available. The task of inference is to compute *P*(**Y**|**X** = **x**), the posterior distribution of the query variables **Y**, conditioned on **X** = **x**. In this paper, we use the algorithm in Lauritzen and Spiegelhalter (1988) to solve the inference problem.

### 2.2. Bayesian multisource data integration

The basic idea of BAMDI is as follows. In our data generation model, we imagine that there exist various brain states that generate a variety of neuroimaging data features. For example, being in one state or another state determines the pattern of functional connectivity in regional resting-state fMRI time courses. To model brain-behavior relationships, we assume that brain states (i.e., “factors”) cause a particular behavioral disposition that is reflected in behavioral measures or scores. That is, the brain states are the parent nodes of behavioral states which can be measured by behavioral variables. There can be many different kinds of brain states that may, or may not, interact in causing a particular behavioral state. Similarly, a particular behavioral state can be caused by one or more brain states. The problem then is to identify the brain-behavior associations in terms of the structure of a Bayesian network. This is accomplished using Bayesian network structure learning, following the identification of brain states using a clustering algorithm.

**Figure 1:**
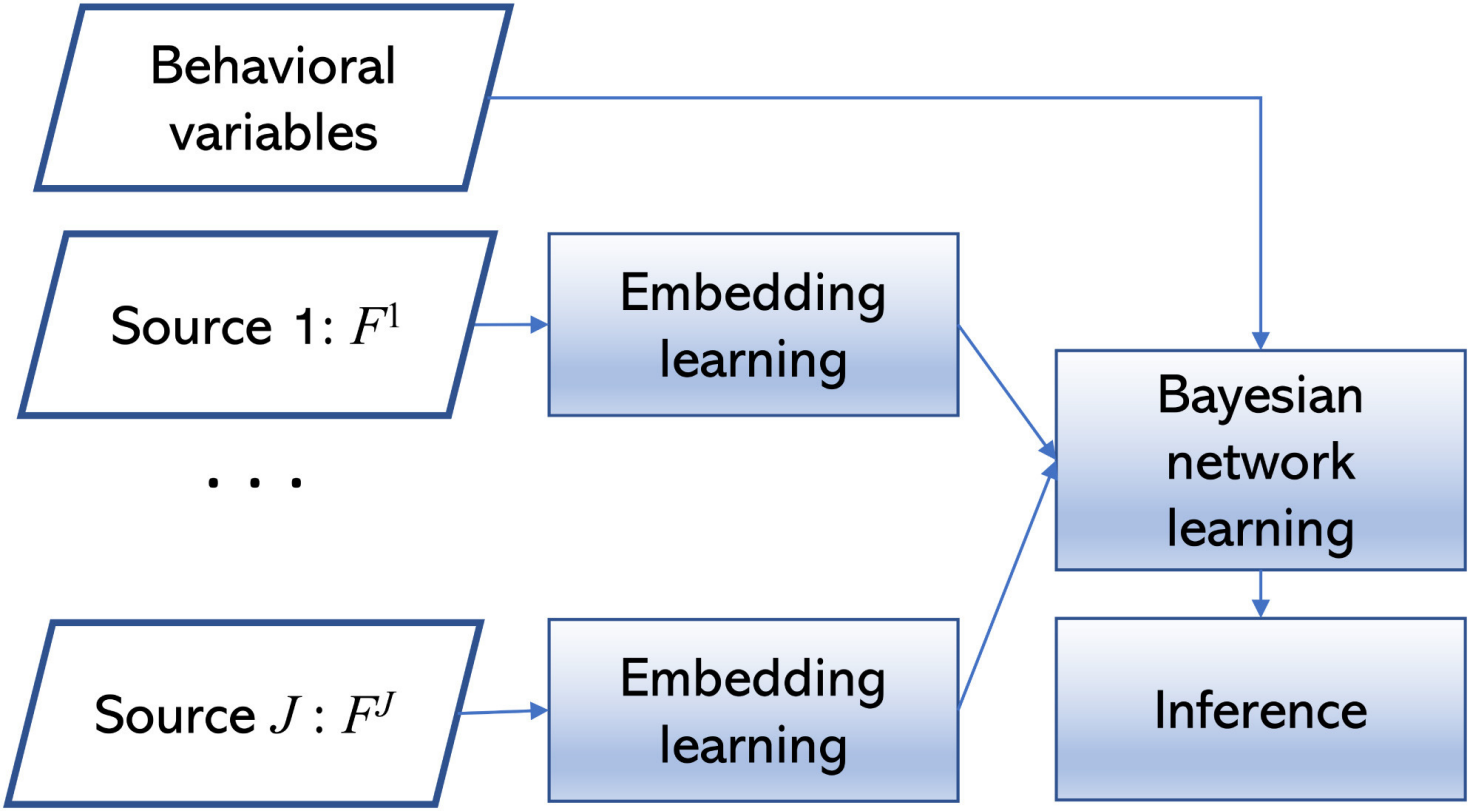
The BAMDI algorithm.

BAMDI learns a Bayesian network B from the observed data **D**. It includes these main modules: embedding learning, Bayesian network learning, and inference. The algorithm is depicted in Figure 1. For source *j*, the feature set **F**^*j*^ is a vector with dimension |**F**^*j*^|, where |**F**^*j*^| is the cardinality of **F**^*j*^. For a study with *I* subjects, the observed data **S**^*j*^ is an *I*×|**F**^*j*^| data matrix. For a study with *J* data sources and *K* behavioral variables, the whole dataset includes {**F**^1^, …, **F**^*J*^} and the associated behavioral variables **B** = {*B*_1_, …, *B*_*K*_}.

The first module is embedding learning. For each data source, we use graph-based clustering to generate an embedding. For **S**^*j*^, we group subjects into clusters. We normalize variables in **F**^*j*^ to zero-mean and unit variance. For subjects *i*_1_ and *i*_2_, we calculate the Euclidean distance *d*_*i*_1_, *i*_2__ and obtain the similarity score as 1/(1+*d*_*i*_1_, *i*_2__). For a study with *I* subjects, this step generates an *I*×*I* similarity matrix that can be treated as a weighted graph. Then we use the multi-level modularity optimization algorithm (Blondel et al., 2008) to detect community structures in the weighted graph. The number of communities is determined by the algorithm. If subjects *i*_1_ and *i*_2_ belong to the same community, they are in the same cluster. Clustering generates a partition of the subject space. We convert this categorical variable into the embedding with one-hot encoding. Each cluster is associated with a binary variable that represents whether a subject belongs to the cluster (0—no, 1—yes). We use $C_{l}^{j}$ to denote the *l*^*th*^ factor of the embedding for source *j*. ${\text{C}}^{j}=\left\{C_{1}^{j},\ldots ,C_{L}^{j}\right\}$. For example, if the clustering algorithm generates 5 clusters, then the embedding contains 5 binary factors.

The second module is Bayesian network learning. We construct a Bayesian network B to describe interactions among {**C**^1^, …, **C**^*J*^, **B**}. We use Bayesian network classifier with inverse-tree structure (BNCIT) to solve this problem (Chen and Herskovits, 2005a,b). BNCIT is an efficient Bayesian network learning algorithm. In BNCIT, the parent set of a node in **B** is a subset of {**C**^1^, …, **C**^*J*^}. There are no edges from **B** to {**C**^1^, …, **C**^*J*^}. We adopt this kind of Bayesian network structure because we focus on studying how the embedding will affect behavioral variables. For a node *X* in **B**, we search for a subset **C**^*s*^ of {**C**^1^, …, **C**^*J*^} which can maximize the BDeu score for structure **C**^*s*^→*X*. That is, the parent set of *X* is determined by ${\text{C}}^{*}={argmax}_{{\text{C}}^{s}}BDeu\left({\text{C}}^{s}\to X\right)$. This search process runs in a node-by-node fashion. After structure learning, the parameters are estimated by the maximum a posteriori method.

The inference module centers on explaining the generated model. The Bayesian network structure reveals important brain-behavior patterns. If the parent set of a behavioral variable includes factors from different data sources, then these sources are synergistic regarding this behavioral variable. If two behavioral variables have shared parent nodes, then these two behavioral variables have a shared brain mechanism. If the factors from a specific data source *j* are not associated with any behavioral variables, then source *j* provides little information about behaviors or source *j* is redundant.

A factor is a binary variable. We use two scores, divergence and mode change, to quantify how the change of factor *C*'s state influences the marginal distribution of behavioral variable *B* by comparing *P*(*B*|*C* = 0) and *P*(*B*|*C* = 1). Both *P*(*B*|*C* = 0) and *P*(*B*|*C* = 1) are discrete probability distributions. We calculate the Jensen–Shannon divergence which is a symmetrized and smoothed version of the Kullback–Leibler divergence (Lin, 1991). For distributions *p* and *q*, the Kullback–Leibler divergence is defined as $D_{KL}\left(p∥q\right)=\sum p\log\frac{p}{q}$. The Jensen–Shannon divergence is defined as *D*_*KL*_(*p*∥*m*)+*D*_*KL*_(*q*∥*m*), where *m* = (*p*+*q*)/2 and *D*_*KL*_(*p*∥*m*) is the Kullback–Leibler divergence between *p* and *m*. The Jensen–Shannon divergence is between 0 (identical) and 1 (maximally different) when the base 2 logarithm is used. For mode change, if the mode of *P*(*B*|*C* = 0) is different from that of *P*(*B*|*C* = 1), the value of this score is 1; otherwise, it is 0.

## 3. Results

### 3.1. Simulated data

We generated simulated data with three data sources (M1, M2, and M3) and four behavioral variables (*BV*_1_, *BV*_2_, *BV*_3_, *BV*_4_). Sources M1, M2, and M3 included 10, 10, and 30 variables, respectively. Source M1 included 2 clusters: samples 1–50 and 151–200 were sampled from a multivariate Gaussian distribution with mean = {3, …, 3} and samples 51–150 were sampled from a multivariate Gaussian distribution with mean = {8, …, 8}. Source M2 included 2 clusters: samples 1–150 were sampled from a multivariate Gaussian distribution with mean = {15, …, 15} and samples 151–200 were sampled from a multivariate Gaussian distribution with mean = {18, …, 18}. For source M3, all samples (1–200) were generated from a multivariate Gaussian distribution with mean = {2, …, 2}.

Let *M*_1_ be a categorical variable to represent the cluster structure of source M1. *M*_1_ = 0 for samples 1–50 and 151–200 and *M*_1_ = 1 for samples 51–150. *M*_2_ = 0 for samples 1–150 and *M*_2_ = 1 for samples 151–200. *BV*_1_ was a noisy version of *M*_1_ with flipping noise 0.1. *BV*_2_ was a noisy version of *M*_2_ with flipping noise 0.1. *BV*_3_ was a noisy version of [*M*_1_ OR *M*_2_]. *BV*_4_ was randomly sampled from {0, 1} and was not associated with *M*_1_ or *M*_2_. *M*_3_ and *BV*_4_ were isolated variables. *M*_3_ was not associated with any behavioral variables and *BV*_4_ was not associated with any sources. We included them to assess whether BAMDI can handle isolated sources and behavioral variables.

**Figure 2:**
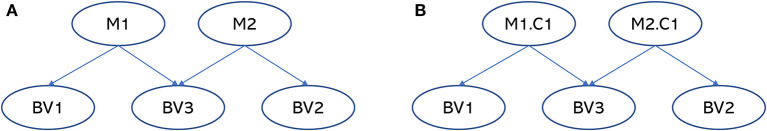
The Bayesian networks for the simulated data. **(A)** Is the ground-truth Bayesian network model to generate the simulated data and **(B)** is the Bayesian network generated by BAMDI. In the ground-truth model, *BV*_1_ is associated with *M*_1_, *BV*_2_ is associated with *M*_2_, and *BV*_3_ is associated with both *M*_1_ and *M*_2_. In the model generated by BAMDI, *M*1.*C*1 is factor 1 from source M1. *M*2.*C*1 is factor 1 from source M2. Other factors were not associated with any behavioral variables and were not shown in the figure. The model generated by BAMDI matches the ground-truth model perfectly.

BAMDI detected two, two, and four clusters for sources M1, M2, and M3, respectively. There were eight factors in the generated embedding (two of them from M1, two of them from M2, and four of them from M3). Figure 2 is the generated Bayesian network. In this figure, *M*1.*C*1 is factor 1 from source M1. *M*2.*C*1 is factor 1 from source M2. Among these factors, two of them (*M*1.*C*1 and *M*2.*C*1) were associated with some behavioral variables. Other factors were not associated with any behavioral variables and were not shown in the figure. *BV*_4_ was not associated with any factors and was not shown in the figure. There are important brain-behavior patterns that can be elucidated from the Bayesian network. First, the Bayesian network revealed that *BV*_1_ was associated with source M1, *BV*_2_ was associated with source M2, and *BV*_3_ was associated with sources M1 and M2. This is expected. Second, *BV*_1_ and *BV*_3_ had a shared brain mechanism because *M*1.*C*1 was a common parent node. *BV*_2_ and *BV*_3_ had a shared brain mechanism because *M*2.*C*1 was a common parent node. Third, sources M1 and M2 were synergistic regarding *BV*_3_ because *M*1.*C*1 and *M*2.*C*1 were jointly predictive of *BV*_3_.

### 3.2. The Adolescent Brain Cognitive Development study

In this experiment, participant data were obtained from the baseline Adolescent Brain Cognitive Development (ABCD) study (release 3.0). 11875 youth (baseline 9–11 years of age) were recruited. Written informed consents were obtained from all parents. All children provided assent to a research protocol approved by the institutional review board at each study site. Details of ABCD MRI acquisition and sequence parameters are in Casey et al. (2018).

Our analysis included these MRI modalities: DTI and resting-state fMRI (rs-fMRI). For DTI, the ABCD database provides a variable for imaging quality. Low quality DTI data were excluded from our analysis. For DTI, standard measures related to white matter microstructural tissue properties were calculated. We used Fractional Anisotropy (FA) which is a measure of the degree of anisotropic water diffusion within a region. FA was averaged across voxels within the Destrieux region-of-interest (ROI) of sub-adjacent white matter. This process generated 148 features (2 hemispheres × 74 regions). The average measures for white matter voxels in the left hemisphere, right hemisphere, and whole brain were also calculated to represent global effects. There were 151 DTI-derived features. To remove batch effects, we used the ComBat algorithm (Fortin et al., 2018) to harmonize these DTI features.

Head motion is a major problem in rs-fMRI and leads to spurious findings. For a 4D rs-fMRI volume, the ABCD database provides information about the total number of frames and the number of frames with low motion. We generated a quality score for motion that was defined as the number of frames with low motion divided by the total number of frames. The quality score was used as an indicator of the overall motion level. We selected subjects with at least half of the frames without excessive head motion (the quality score of motion > 0.5). We excluded subjects with incomplete data (those with missing values).

For rs-fMRI, the imaging-derived features were correlation between distributed networks of brain regions (Marek et al., 2019). Thirteen brain networks were detected, including auditory network (“ad”), cingulo-opercular network (“cgc”), cingulo-parietal network (“ca”), default network (“dt”), dorsal attention network (“dla”), frontoparietal network (“fo”), “none” network (“n”), retrosplenial temporal network (“rspltp”), sensorimotor hand network (“smh”), sensorimotor mouth network (“smm”), salience network (“sa”), ventral attention network (“vta”), and visual network (“vs”) (Gordon et al., 2017). Notice that these brain networks comprised ROIs with positive correlations, which means that the average signal reflects the activity of the network. Each network was treated as a node. Functional connectivity between node A and node B was measured by calculating the correlation coefficient between the average signal of A and that of B. There were 78 rs-fMRI-derived features. Each feature represented functional connectivity between a brain network pair.

In the ABCD study, the NIH Toolbox cognition measures were used to assess child cognition (Luciana et al., 2018). The seven cognitive tasks in the NIH Toolbox included the dimensional change card sort task to assess cognitive flexibility (“cardsort”), list sorting working memory task to assess working memory (“list”), picture sequence memory task to assess episodic memory (“picture”), pattern comparison processing speed task to assess processing speed (“pattern”), picture vocabulary task to measure vocabulary comprehension (“picvocab”), oral reading recognition task to measure language/reading decoding (“reading”), and the flanker task to assess attention and inhibition (“flanker”). The neurocognitive battery was administrated using an iPad with one-on-one monitoring by a research assistant. The total time for administration was about 35 min. Based on the seven task scores, three composite scores were generated: a total score composite (“totalcomp”), a crystallized intelligence composite (“cryst”), and a fluid intelligence composite (“fluidcomp”). The age-corrected total score composite has a mean of 100 and a standard deviation of 15. For measures of cognition, higher scores represented better cognitive ability. The age-corrected scores were used as the behavioral variables in this study. These behavioral variables were binarized based on the sample median.

**Figure 3:**
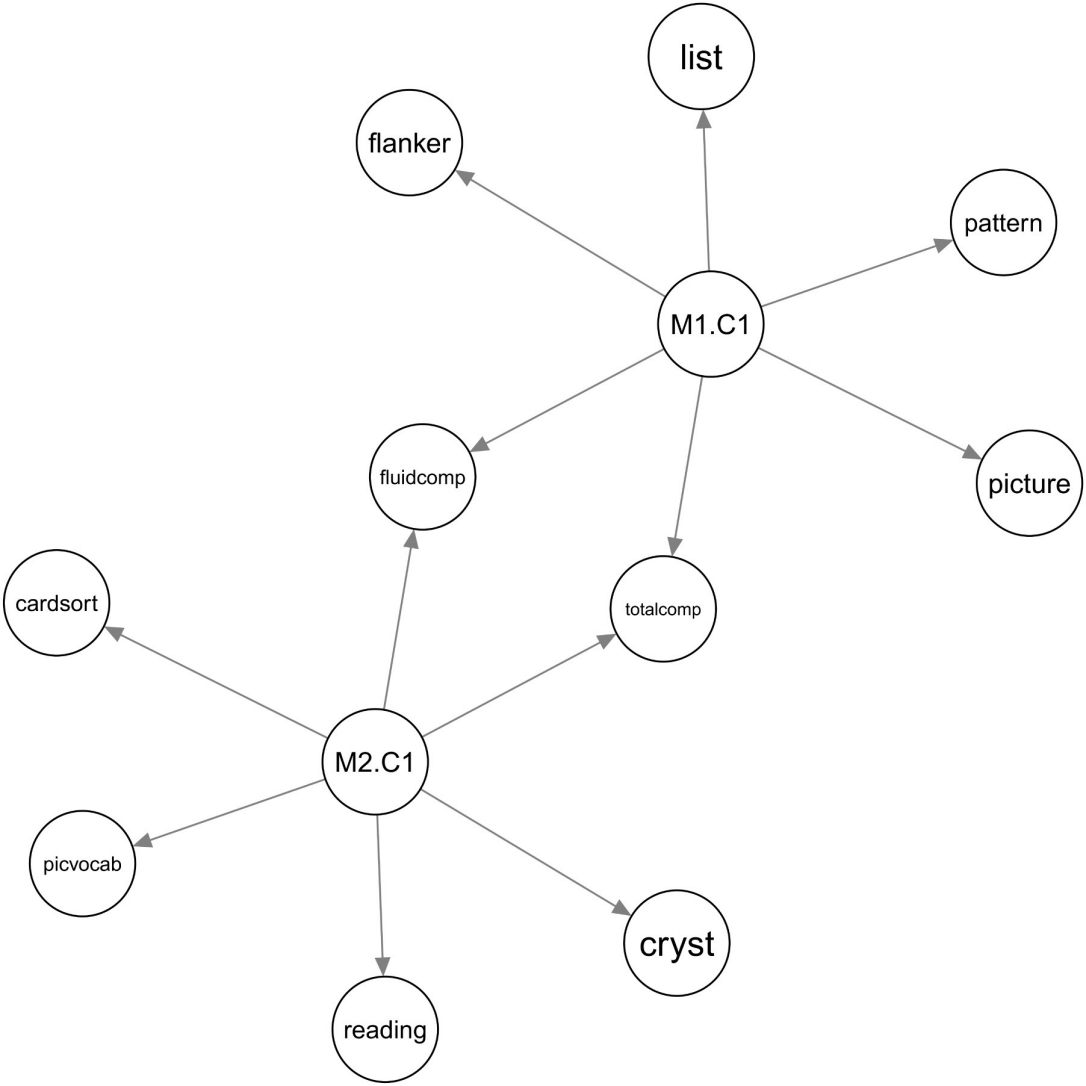
The Bayesian network for the ABCD study. Source M1 is DTI and source M2 is rs-fMRI. *M*1.*C*1 is factor 1 from DTI. *M*2.*C*1 is factor 1 from rs-fMRI. Other factors were not associated with any behavioral variables and were not shown in the figure.

For DTI (source 1), BAMDI generated two factors. For rs-fMRI (source 2), BAMDI generated three factors. Among these five factors, two of them were associated with behavioral variables (Figure 3). DTI and rs-fMRI were synergistic regarding the fluid intelligence composite and the total score composite. The list sorting, flanker, picture sequence memory, and pattern comparison processing speed tasks were associated with DTI. The dimensional change card sort, picture vocabulary, oral reading recognition tasks, and crystallized intelligence composite were associated with rs-fMRI.

**Figure 4:**
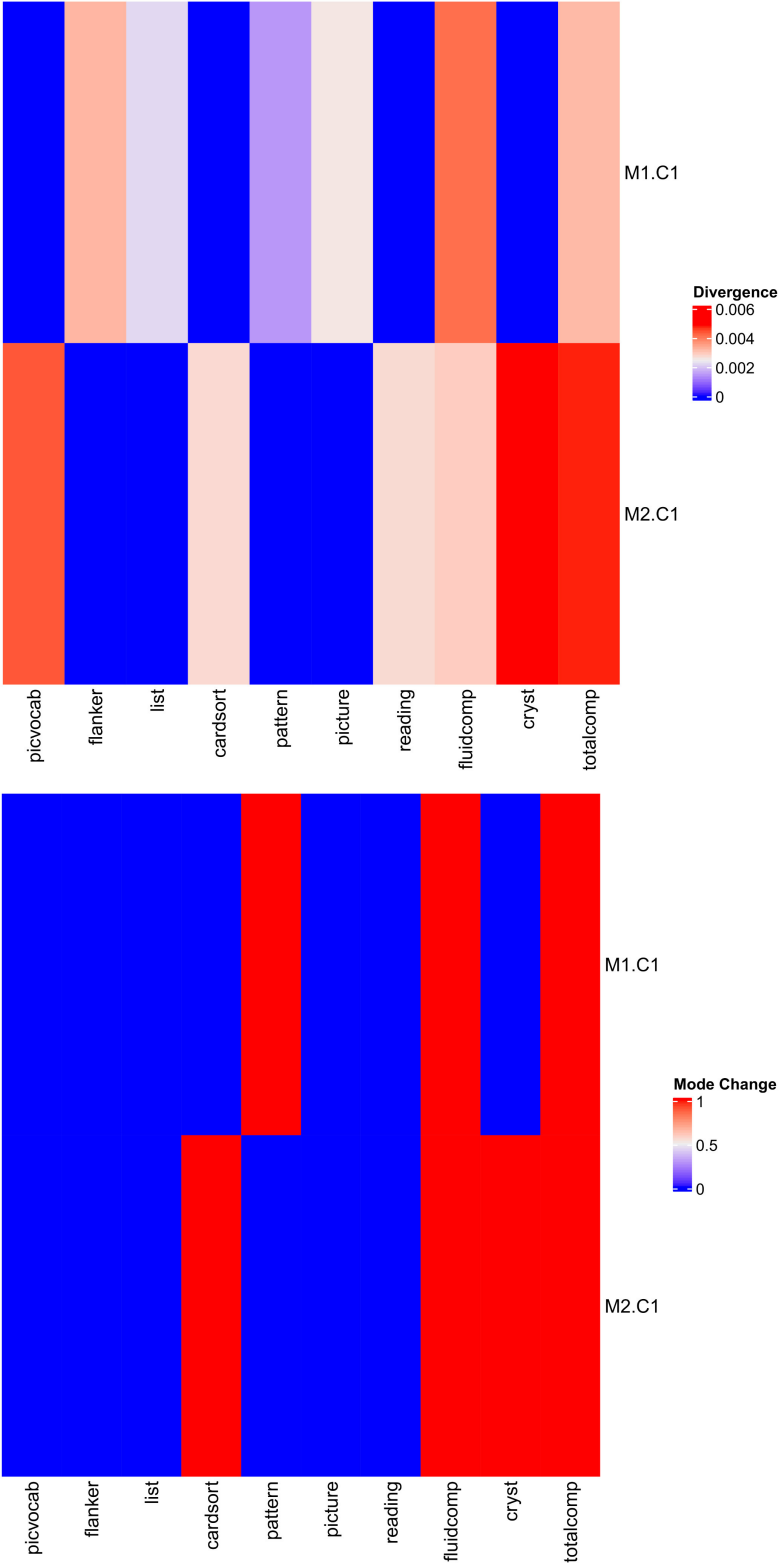
The divergence and mode change score for the ABCD study.

The divergence and mode change score are depicted in Figure 4. *M*1.*C*1 (factor 1 from DTI) had high divergence and high mode change score for the fluid intelligence composite and total score composite. That is, the change of *M*1.*C*1 changed the posterior marginal distribution of the fluid intelligence composite and total score composite. *M*2.*C*1 (factor 1 from rs-fMRI) had high divergence and high mode change score for the fluid intelligence composite, total score composite, and crystallized intelligence composite. That is, the change of *M*2.*C*1 changed the posterior marginal distribution of the fluid intelligence composite, total score composite, and crystallized intelligence composite.

**Figure 5:**
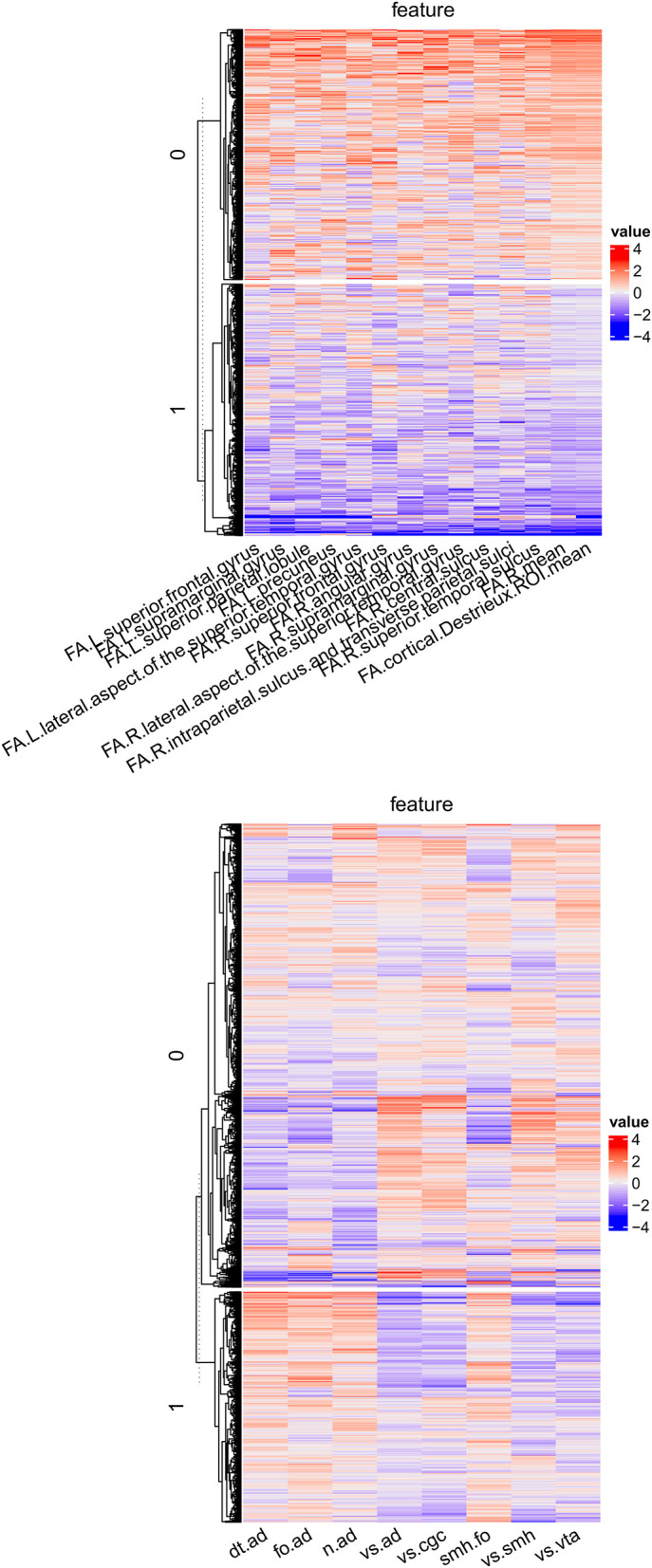
The imaging markers for DTI and rs-fMRI based factors.

To annotate important factors, we detected imaging markers to characterize factors. For a factor *C*^*j*^ from source *j*, we performed analysis of variance (ANOVA) with an imaging feature *F*^*j*^ as the dependent variable and *C*^*j*^ as the independent variable. Then we ranked imaging features based on the effect size and selected the top 10% features as the imaging markers. The imaging markers are shown in Figure 5. For DTI, the factor *M*1.*C*1 represented a subtype that had lower FA in the whole brain, right hemisphere, left superior frontal gyrus, left supramarginal gyrus, left superior parietal lobule, left precuneus, left lateral aspect of the superior temporal gyrus, right superior frontal gyrus, right angular gyrus, right supramarginal gyrus, right lateral aspect of the superior temporal gyrus, right central sulcus, right intraparietal sulcus and transverse parietal sulci, and right superior temporal sulcus. For rs-fMRI, the factor *M*2.*C*1 represented a subtype that had higher functional connectivity between the default network and auditory network, frontoparietal network and auditory network, “none” network and auditory network, sensorimotor hand network and frontoparietal network, and lower functional connectivity between visual network and auditory network, visual network and cingulo-opercular network, visual network and sensorimotor hand network, and visual network and ventral attention network.

## 4. Discussion

Data fusion is important for the understanding of inter-dependencies and relations across heterogeneous types of data. We propose a data fusion method called BAMDI to model the interactions among data sources and behavioral variables. The generated Bayesian network describes brain-behavior relationships. It is explainable: (1) the structure of Bayesian network reveals important brain-behavior patterns such as source synergy; (2) the divergence and mode change score assess how the change of factor affects the marginal distribution of behavioral variables.

We assessed the performance of BAMDI in two studies: simulated data and the ABCD study. For the simulated data, BAMDI correctly detected the brain-behavior patterns including *BV*_3_ is a noisy version of [*M*_1_ OR *M*_2_]. For the ABCD study, the two data sources, DTI and rs-fMRI, were synergistic regarding the fluid intelligence composite and the total score composite. The change of *M*1.*C*1, a DTI-derived factor that was characterized by lower FA in many regions, changed the posterior marginal distribution of the fluid intelligence composite and total score composite. The change of *M*2.*C*1, a rs-fMRI derived factor characterized by hyper-connectivity related to the auditory network and hypo-connectivity related to the visual network, changed the posterior marginal distribution of the fluid intelligence composite, total score composite, and crystallized intelligence composite.

Data integration methods can be classified into three different categories: early integration, intermediate integration, and late integration. Early integration focuses on combining data before applying a learning algorithm. An example of early integration is learning a common latent representation. Intermediate integration produces a joint model learned from different sources simultaneously. Late integration methods model different sources separately, then combines the outputs. BAMDI is a late integration method. BAMDI is also related to collective learning. Collective learning (Chen et al., 2004) is a machine learning framework to learn a model from multiple and diverse datasets by stage-wise learning (local learning and cross learning). Under this framework, the embedding learning step in BAMDI is local learning and the Bayesian network learning step in BAMDI is cross learning.

One of the limitations of BAMDI is that it requires discrete behavioral variables. Some behavioral variables such as disease diagnosis (normal controls or Alzheimer's disease) are naturally discrete; while others may be continuous. For continuous behavioral variables, we need to discretize them and this discretization process may cause a loss of information. We could extend BAMDI to handle continuous behavioral variables. In this extension, we adopt the conditional Gaussian Bayesian network representation and the local distribution *P*(*X*|*pa*(*X*)) is a Gaussian mixture. This will be the focus of our future work.

## Data availability statement

Publicly available datasets were analyzed in this study. This data can be found at: https://abcdstudy.org/.

## Ethics statement

The ABCD study was approved by the ABCD Site Ethics Committee. A listing of participating sites and a complete listing of the study investigators can be found at https://abcdstudy.org/principal-investigators/. The patients/participants provided their written informed consent to participate in this study.

## Author contributions

RC designed the study, implemented the algorithm, conducted the experiments, and wrote the manuscript.

## Funding

This work was partially supported by the NIH NINDS and the BRAIN Initiative (R01NS110421) and NIH NIDA (UG3DA053802).

## Conflict of interest

The author declares that the research was conducted in the absence of any commercial or financial relationships that could be construed as a potential conflict of interest.

## Publisher's note

All claims expressed in this article are solely those of the authors and do not necessarily represent those of their affiliated organizations, or those of the publisher, the editors and the reviewers. Any product that may be evaluated in this article, or claim that may be made by its manufacturer, is not guaranteed or endorsed by the publisher.

## Author disclaimer

This manuscript reflects the views of the authors and may not reflect the opinions or views of the NIH or ABCD consortium investigators.
